# Incidence Rates and Deaths of Tuberculosis in HIV-Negative Patients in the United States and Germany as Analyzed by New Predictive Model for Infection

**DOI:** 10.1371/journal.pone.0042055

**Published:** 2012-10-15

**Authors:** Yudong Ren, Fan Ding, Siqingaowa Suo, Ri-e Bu, Dante S. Zarlenga, Xiaofeng Ren

**Affiliations:** 1 College of Veterinary Medicine, Northeast Agricultural University, Harbin, China; 2 School of Life Science, Inner Mongolia University for the Nationalities, Tongliao, Inner Mongolia, China; 3 Department of Health, Chinese Armed Police forest Force Command, Beijing, China; 4 Animal Parasitic Diseases Laboratory, Agricultural Research Service, Beltsville, Maryland, United States of America; Commissariat a l'Energie Atomique(cea), France

## Abstract

Incidence and mortality due to tuberculosis (TB) have been decreasing worldwide. Given that TB is a cosmopolitan disease, proper surveillance and evaluation are critical for controlling dissemination. Herein, mathematical modeling was performed in order to: 1) demonstrate a correlation between the incidence of TB in HIV-free patients in the US and Germany, and their corresponding mortality rates; 2) show the utility of the newly developed D-R algorithm for analyzing and predicting the incidence of TB in both countries; and 3) inform us on population death rates due to TB in HIV-negative patients. Using data published by the World Health Organization between 1990 and 2009, the relationship between incidence and mortality that could not be ascribed to HIV infection was evaluated. Using linear, quadratic and cubic curves, we found that a cubic function provided the best fit with the data in both the US (Y = 2.3588+2.2459X+61.1639X^2^−60.104X^3^) and Germany (Y = 1.9271+9.4967X+18.3824X^2^−10.350X^3^) where the correlation coefficient (R) between incidence and mortality was 0.995 and 0.993, respectively. Second, we demonstrated that fitted curves using the D-R model were equal to or better than those generated using the GM(1,1) algorithm as exemplified in the relative values for *Sum of Squares of Error*, *Relative Standard Error*, *Mean Absolute Deviation*, *Average Relative Error*, and *Mean Absolute Percentage Error*. Finally, future trends using both the D-R and the classic GM(1,1) models predicted a continued decline in infection and mortality rates of TB in HIV-negative patients rates extending to 2015 assuming no changes to diagnosis or treatment regimens are enacted.

## Introduction

Tuberculosis (TB) is highly contagious and in particular, when one's immune system has been previously compromised. Co-infection with TB bacilli and HIV can be lethal. An HIV-positive individual infected with TB bacilli is more likely to become sick than one who is TB-positive and HIV-negative (http://www.who.int/mediacentre/factsheets/fs104/en/). It has been reported that 5–10% of this subset of individuals (TB-positive HIV-negative) become sick or infectious at some point in their lifetimes. (http://www.who.int/mediacentre/factsheets/fs104/en/).

Given that TB is a cosmopolitan disease, proper surveillance and evaluation are critical for controlling its spread [1], [2]. Control and treatment of TB have been studied in recent years by numerous labs [3], [4]. Research has been performed on the relationship between genome sequencing and epidemiology [5], associations between mutation and infectivity [6], surveillance on sub-populations such as children and immigrants a [7], [8], and surveillance and treatment predicated upon computer assisted detection [9] among others. The TB control program DOTS, launched by the World Health Organization (WHO) in 1995, has led to efficient treatment for 41 million patients. In 2006, a new and more aggressive WHO sponsored program was implemented according to The Global Plan to Stop TB, 2011–2015 (http://www.stoptb.org/global/plan/). Understanding and being able to predict the future progression of this disease is important for evaluating current control measures if the overall goal of eradication is to be met. Herein, we were able to show a strong correlation between TB infection and mortality that was independent of HIV-infection suggesting that HIV is not the only contributing factor to mortality in TB- patients. Furthermore, we demonstrated the utility of the D-R algorithm for analyzing the incidence and mortality in TB-infected, HIV-negative patients in the US and in Germany as a proof of principle. We then used this modeling process to extrapolate future trends in both rates through 2015 assuming no changes in treatment or prevention. We propose that these data provide a baseline to which we can reference changes in TB infection and mortality that may result from enacting and implementing the new WHO “Stop TB Strategy”.

## Materials and Methods

### Data collection and analysis

Data on the incidence (I-U) and mortality (D-U) of TB in the US, and incidence (I-G) and mortality (D-G) of TB in Germany among HIV-negative people between 1990 and 2009 were collected from the official website of the WHO [10]. Correlation coefficients and curve fitting were used to investigate the interrelationship between the incidence and death due to TB in HIV-negative patients in the both countries. The data was evaluated using linear, quadratic and cubic functions to obtain the method which would provide the best fit with the data. The statistical analyses were performed by trend χ2 test using SPSS13.0 software.

Secondly, the incidence of TB, and the numbers of deaths in TB-infected HIV-negative patients in both countries were independently simulated using D-R and GM(1,1) models [11]–[13]. To calculate this, the first order differential of the mortality data at each point was analyzed using the D-R model. The weighting of the differences in first order values were considered to be the short term trends. Then the mean values of the difference in first order values were used to generate the long-term trends. Predicted values were then generated from both the short-term and long-term trends as well as the weighting of short-term data used to initiate the analysis. Generally the weighting of short-term trend was higher. The above-mentioned cycle was repeated continuously to get the predicated mortality values.

Comparisons between the actual values and values derived from each algorithm were analyzed using the Sum of squares of error (SSE), Relative standard error (RSE), Mean absolute deviation (MAD), Average relative error (ARE), and the Mean absolute percentage error (MAPE) [11], [12], [14] to test the accuracy of each simulation beginning with year 1994 and extending through 2009 (note: years 1990–1993 were required to develop the equations for simulation and therefore not included in data analysis). Finally, once the most predictive equations were developed, the incidence and death rates of TB in HIV-negative patients were extrapolated for the years 2010 through 2015 in both countries.

## Results

### Statistical analyses

Data on I-U, D-U, I-G and D-G between 1990 and 2009 are summarized in Table 1. Four groups of data evaluated by χ^2^ test (*(trendχ^2^ test)*) had a p<0.01 indicating that the data was significant and that the incidence and death rates have decreased annually.

**Table 1:** Actual (At) and simulated (D-R & GM(1,1)) values for the incidence (I) and death (D) caused by tuberculosis in the US (U) and Germany (G).

Year	I-U			I-G			D-U			D-G		
	At	D-R	GM	At	D-R	GM	At	D-R	GM	At	D-R	GM
1990	13.00			23.00			0.60			1.30		
1991	13.00			21.00			0.59			1.10		
1992	12.00			21.00			0.59			1.20		
1993	11.00			21.00			0.50			1.10		
1994	11.00	10.21	10.13	19.00	20.96	24.00	0.47	0.43	0.48	0.94	1.03	1.50
1995	9.70	10.92	10.07	17.00	17.53	19.07	0.42	0.44	0.44	0.86	0.82	0.95
1996	8.90	8.73	9.23	17.00	15.41	17.05	0.39	0.38	0.39	0.85	0.79	0.84
1997	8.20	8.24	8.45	16.00	16.84	16.21	0.36	0.36	0.36	0.80	0.83	0.79
1998	7.50	7.61	7.76	15.00	15.23	15.33	0.32	0.34	0.33	0.73	0.76	0.74
1999	7.10	6.93	7.11	14.00	14.21	14.44	0.31	0.29	0.30	0.72	0.68	0.69
2000	6.50	6.75	6.59	13.00	13.19	13.56	0.28	0.30	0.28	0.67	0.71	0.65
2001	6.30	6.02	6.08	9.70	12.19	12.69	0.28	0.26	0.25	0.43	0.63	0.62
2002	5.90	6.11	5.68	9.70	7.20	11.26	0.25	0.28	0.24	0.50	0.25	0.53
2003	5.70	5.58	5.31	9.10	9.48	10.25	0.24	0.23	0.22	0.46	0.54	0.49
2004	5.60	5.53	5.00	8.40	8.59	9.38	0.23	0.23	0.21	0.42	0.43	0.45
2005	5.30	5.51	4.74	7.70	7.85	8.60	0.23	0.22	0.19	0.39	0.39	0.41
2006	5.20	5.07	4.50	7.00	7.13	7.89	0.22	0.23	0.18	0.35	0.36	0.38
2007	5.00	5.11	4.29	6.40	6.43	7.24	0.22	0.21	0.17	0.34	0.32	0.35
2008	4.80	4.84	4.10	5.10	5.90	6.64	0.21	0.22	0.17	0.23	0.33	0.32
2009	4.10	4.64	3.92	4.90	4.10	6.02	0.16	0.20	0.16	0.23	0.15	0.29
2010		3.57	3.69		4.66	5.48		0.12	0.15		0.22	0.26
2011		3.14	3.46		4.45	5.07		0.09	0.14		0.22	0.24
2012		2.77	3.24		4.28	4.70		0.07	0.13		0.21	0.22
2013		2.47	3.04		4.14	4.36		0.05	0.12		0.21	0.20
2014		2.22	2.85		4.02	4.04		0.03	0.11		0.20	0.19
2015		2.01	2.67		3.92	3.74		0.01	0.10		0.20	0.17

### Relationship between the incidence and death of HIV-negative TB patients

Using SPSS13.0, the coefficient (R) relating incidence (I-U) and death (D-U) in the United States was 0.997 (p<0.01) indicating a close link between the two sets of data. In like manner, the association between the incidence (I-G) and death (D-G) in Germany were equally high (R = 0.993,p<0.01). Based on these results, linear, quadratic and cubic parametric functions were used to evaluate the relationships between incidence and mortality in the US and Germany to determine the best fit. Resulting curves showed that a cubic function was most consistent with the existing data. In this regard, the following equations for the US and Germany, respectively, were generated; Y = 2.3588+2.2459X+61.1639X^2^−60.104X^3^ and Y = 1.9271+9.4967X+18.3824X^2^−10.350X^3^. Using these equations, the calculated R values were 0.995 and 0.993, respectively (Table 2). In addition, the variance (F) was the smallest (p<0 .01) when using a cubic equation relative to linear and quadratic equations (Table 2). In developing these equations the dependent variables were defined by I-U and I-G.

**Table 2:** The correlation (R) and variance (F) between incidence (I) and death (D) in TB patients in the US (U) and Germany (G) using linear, quadratic, and cubic parametric equations.

	I-U and D-U		I-G and D-G	
	R	F	R	F
Linear	0.990	1849.79	0.986	1262.96
Quadratic	0.992	1115.52	0.993	1137.90
Cubic	0.994	863.39	0.995	1040.75

For F, p<0.01.

### Relationship between actual and calculated values for the incidence and death of HIV-negative, TB patients

The incidence and death of HIV-negative TB patients in the US and Germany were calculated using the D-R and GM(1,1) models. As shown in Table 1, the values predicated on the D-R algorithm were equal to or better than those generated by the GM(1,1) model when compared to actual values.

**Figure 1:**
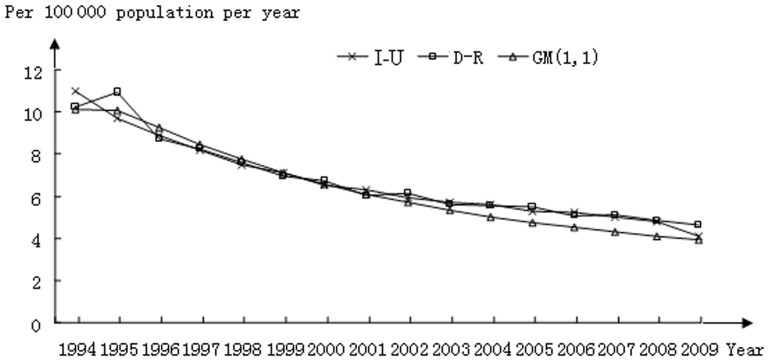
Incidence of tuberculosis in the US; a comparison between actual (I-U) and calculated datasets using the D-R and GM(1,1) models.

Comparisons between I-U calculations derived from both models are shown in Figure 1. As one can see, in the earlier years (1996–2001) the actual data and the fitted curves derived from both algorithms are superimposed; however, beginning in year 2002, the curve derived from the GM (1,1) model diverged from the actual data while that derived from the D-R algorithm continued aligning with the actual dataset.

**Figure 2:**
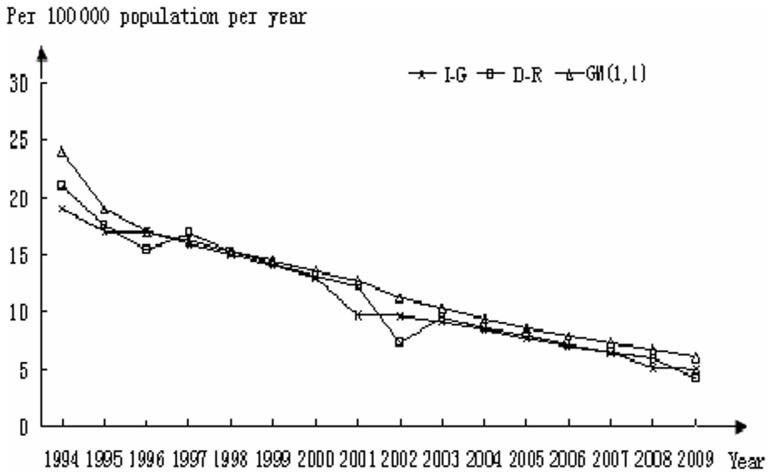
Incidence of tuberculosis in Germany; a comparison between actual (I-G) and calculated datasets using the D-R and GM(1,1) models.

In Figure 2, comparisons among actual and calculated values for I-G showed good alignment except for the years 1994–1996 and an anomaly that surfaced in 2001 and 2002. In both cases, the D-R and GM(1,1) models deviated from the actual dataset; however, the D-R algorithm was better able to account for the deviation in linearity that occurred in 2001 and 2002 than the GM(1,1) model.

**Figure 3:**
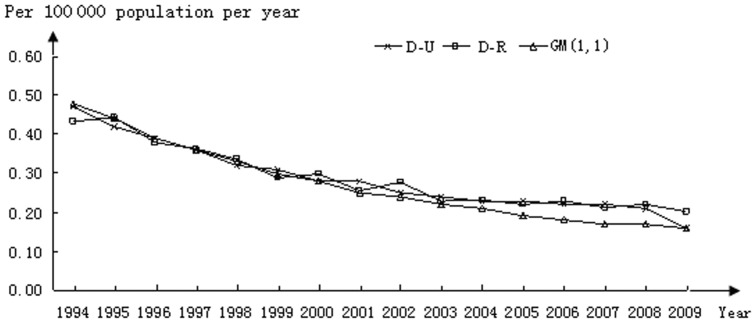
Mortality of HIV-negative TB patients in the US; a comparison between actual (D-U) and calculated datasets using the D-R and GM(1,1) models.

**Figure 4:**
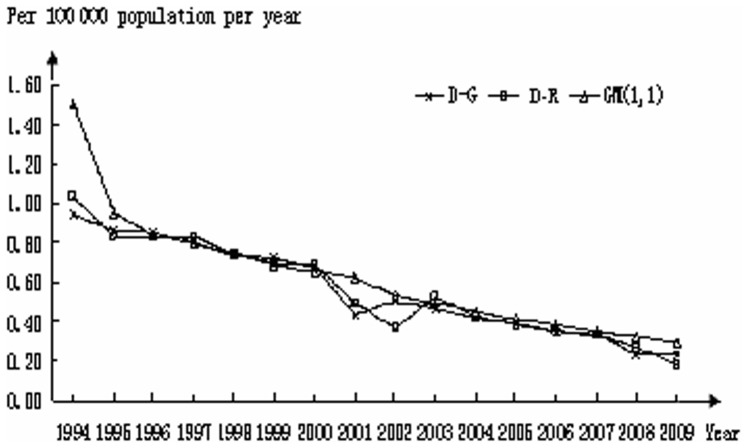
Mortality of HIV-negative TB patients in Germany; a comparison between actual (D-G) and calculated datasets using the D-R and GM(1,1) models.

Comparisons among mortality rates were also evaluated using both models. As shown in Figure 3, both models superimposed over the actual data between 1995 and 2000. In 2001–2002 the curve predicted using the GM(1,1) model began to deviate from the actual dataset which continued throughout the rest of the analysis except for 2009. In like manner, a deviation from the actual data occurred in 2001 and 2002 using the D-R model; however, as with the I-G data, the model was able to self-correct and resulted in good alignment with the actual data through 2008. A deviation from the actual data occurred again in 2009. Analysis of D-G is shown in Figure 4. In most aspects, the profiles mirror those generated for mortality rates in the US with deviations between actual and predicted values occurring in the early years and again in years 2001 and 2002.

### Predictive values of the D-R and GM(1,1) algorithms

From the 2011 WHO report on TB, the actual values for I-U, I-G, D-U and D-G were 4.1, 4.8, 0.18, and 0.25, respectively. The values predicted using the D-R model were 3.6, 4.7, 0.12 and 0.22, respectively. Calculations for the indexes SSE, RSE, MAD, ARE and MAPE derived from the D-R and GM(1,1) algorithms are summarized in Table 3. In general, the values for the D-R model are equal to or less than those generated using the GM(1,1) model suggesting that the D-R algorithm was equal to or better than the GM(1,1) model for predicting trends in TB infection and mortality. These results held true for data derived from both the US and for Germany.

**Table 3:** Calculation of key accuracy indexes spanning 1994–2009 evaluating the predictive quality of the D-R and GM(1,1) models.

	I-U		I-G		D-U		D-G	
	D-R	GM(1,1)	D-R	GM(1,1)	D-R	GM(1,1)	D-R	GM(1,1)
**SSE**	2.76	3.58	21.43	49.54	0.01	0.01	0.04	0.38
**RSE**	0.43	0.49	1.20	1.82	0.02	0.03	0.05	0.16
**MAD**	0.28	0.40	0.81	1.29	0.02	0.02	0.04	0.08
**ARE**	−1.26%	4.65%	−1.50%	−13.22%	−1.70%	6.39%	−0.18%	−12.97%
**MAPE**	3.99%	6.59%	7.93%	13.22%	6.46%	7.65%	7.86%	14.17%

**Note:** I-U, I-G, D-U and D-G represent the incidence (I) and deaths (D) of tuberculosis in the US (U) and Germany (G), among HIV-negative TB patients in the. D-R and GM(1,1) define the algorithms from which these values were derived.

## Discussion

Related coefficient analyses (R) indicated that the incidence of TB and deaths due to TB of HIV-negative patients were closely related in the both countries i.e. U = 0.997, p<0.01 and G = 0.993, p<0.01. Comparisons among different simulation approaches showed that cubic parametric functions generated the best fit between the incidence and mortality in TB-infected, HIV-negative patients.

Actual data clearly showed that the incidence and mortality of TB in HIV-negative patients in the US and Germany decreased during the period 1990–2009. In predicting this trend using existing datasets, the curves generated using the D-R model were equal to or better than those generated by the GM(1,1) model with respect to coinciding with actual datasets except for the last available dataset i.e. 2010. It is possible that this difference lies in the fact that the 2010 dataset included HIV-positive patients in the reporting of incidence. As such it was not included in our consideration of incidence for 2010. In addition, in those instances where rapid points of inflection appeared in the actual data, the self-adaptation component of the D-R model was able to correct within one data point and in stark contrast to the curves generated with the GM(1,1) algorithm. The D-R model consists of weighting first order differential of the original data, weighting the short-and long-term trends, and then summing these with the original data to arrive at a predicted value. The advantage of this approach is that regardless of how the original data may change, the model can adapt to this change resulting in little deviation between the simulated and actual values.

In contrast, the GM(1,1) model mainly relies on summing or totting-up the original data. In other words, rather than summing the arithmetic series i.e., 1+2+3+4 etc., the GM(1,1) model uses accumulated data or a progressive total such as in the series 1,(1+2) 3,(1+2+3) 6, (1+2+3+4), 10,(1+2+3+4+5) 15 etc. to arrive at the predicted values. As the series increases, the totting-up values also increase which in turn reduces the flexibility of the GM(1,1) model. The D-R model has no such drawbacks. In general, we attribute the variability observed in the earlier years (1994–1996) to insufficient data (1990–1993) for simulating that part of the curve. These inconsistencies were accounted for as the numbers of years incorporated in the analysis increased.

As we bring out in this study and confirmed in previous analyses (11), the D-R model can self-adapt. This allows the model to have a high level of flexibility and generate reliable future trends with limited i.e., incomplete, data. We hope to further improve this model by incorporating and weighting a delay operator (a dataset encompassing the differences between the fitted values and the actual values to increase the fitting accuracy of the model) and by adding a limit algorithm to increase the model's predictive character.

Herein we stringently compared our model to the GM(1,1) model because of the wide use of the GM(1,1) model in these types of analyses; however, other models are also commonly used for time series data such as, ARMA and ARIMA. These time series algorithms are more suited for data with high periodicity. For this specific purpose, they may be better than the D-R model. However, when predicting trends involving general increases and/or decreases in the absence of recurring changes such as what occurs with TB and drug-resistance, these models are inferior to the D-R model.

By comparing predicted and actual values, we can conclude that the predictive values of the D-R model were accurate and feasible. In like manner, the predicative values of GM(1,1) model were also accurate and can be used as a reference. Both the actual values and the values predicted by D-R and GM(1,1) models indicated that under the current policies and prevention methods, I-U, I-G, D-U and D-G decreased over the period of the analysis where the trend in D-G was the most significant.

Comparing our method and that used by the WHO i.e., log-linear model, we have concluded that the D-R model is better suited for simulating this data. First, the log-linear model is commonly used for examining “interactions” or the influence between two data sets or parameters, rather than for simulating and predicting future trends derived from one data set. In this manuscript, we first calculated the correlation coefficient, and then performed linear fitting, then curve fitting and additional curve fitting. Finally, we compared the various fitted curves to obtain the best equation that describes the occurrence and death rates of TB and their relationship to one another in US and in Germany. We feel the log-linear model would have been more appropriate had we been interested in comparing the data from Germany and the US with respect to the effects of intervention strategies, for example, rather than predicting future trends. As such, we feel the D-R model is more suitable than the log-linear model for analyzing future trends. Other advantages of using either the D-R or GM models over the log-linear model are the ability to generate exact fitting results and the fact that the D-R or the GM models which are designed for predicting future trends would be helpful for policy formulation and disease control.

We refrained from a specific one-on-one comparison of our model with that used to generate the most recent WHO reports because there was no way to guarantee that our datasets would be 100% congruent. As example, we culled TB patients that harbored HIV infections. Attempting to re-analyze the data used in our report using the log-linear model and generating results not consistent with the WHO report would also bring into question the data sources. As such, this was not done.

The efficiency of the D-R algorithm to mirror actual data was further confirmed and supported by accuracy indexes as defined here by SSE, RSE and MAD which were lower or equal to those generated by the GM(1,1) model. We also evaluated ARE and MAPE which derive their comparisons based on percent differences rather than concrete numbers and as such may be more representative of the differences between the two algorithms. In this regard, the values obtained using the D-R model were consistently lower suggesting a better accuracy than those derived using the GM(1,1) model. Of particular interest are future predictions using D-R model which shows that the incidence and mortality due to TB of HIV-negative patients should continue to decrease in both countries through 2015 (see Table 1). Using SPSS software, we tested the four groups of data by the chi-square trend test. Our results showed that all data sets were statistically-significant (P<0.001) and generated the following values; I-U = 19.834, I-G = 18.979, D-U = 20.028, and D-G = 18.141. The higher value in the D-U data relative to that from D-G suggests a more pronounced reduction in the US death rate due to TB. The results imply that the current control measures have been effective. These data also suggest that D-U will decrease more significantly if the trends continue. As such, we predict that by 2015, the mortality could drop to 1%(0.01)in the US.

Clearly, the ability of our model to “self-correct” needs to be more exhaustively tested and for this reason we evaluated the data related TB in the US and Germany. Certainly, no one model can predict far into the future given that population interactions and environments are continuously changing and can never fully be accounted for. However, if small inflection points can be identified early on and a flexible model is available that can alter its predictive character for the near term as well as the intermediate future, such an algorithm could be a tremendous asset to policy makers, governments and the research and medical communities who have control over short term intervention strategies. We believe the D-R model in one such algorithm with this potential.

In conclusion, we used a cubic parametric equation for the first time to show a relationship between incidence and mortality among HIV-negative TB-infected patients in America and Germany with the hope of using this as a benchmark for predicting future changes. Furthermore, such data allows us to link the incidence of TB to mortality and from this propose that prevention needs to receive more attention as a key approach to reducing mortality rates. In addition, we showed that the D-R model closely mirrored the trend line of TB through 2009 and from this, predicted changes to the infection and mortality rates through 2015.
